# Symmetric Siamese Networks for Longitudinal Chest Radiograph Change Detection: A Leakage-Controlled Study on MIMIC-CXR

**DOI:** 10.3390/tomography12090129

**Published:** 2026-09-09

**Authors:** Şahin Işık, Hakan Alp Eren

**Affiliations:** 1 Department of Computer Engineering, Engineering and Architecture Faculty, Eskişehir Osmangazi University, 26040 Eskişehir, Turkey; 2 Department of Software Engineering, Engineering and Architecture Faculty, Eskişehir Osmangazi University, 26040 Eskişehir, Turkey; hakan.eren@ogu.edu.tr

**Keywords:** chest radiography, change detection, Siamese networks, longitudinal imaging, MIMIC-CXR, data leakage, explainable AI

## Abstract

**Background/Objectives:** Radiologists usually read a chest radiograph by comparing it with an earlier one, yet most deep learning models are trained and tested on single images. This study measures how much a longitudinal pair of radiographs improves finding-specific change detection on MIMIC-CXR and tests whether the gain can be explained by encoder pretraining. **Methods:** One frontal pair per patient was built from the earliest and latest studies within 180 days (14,043 pairs), and paired CheXpert labels defined four transition classes: absent-to-absent, onset, resolved, and persistent. A Siamese DenseNet-121 with two symmetric, weight-sharing branches fused the two images through concatenation and feature difference, and it was compared with a matched baseline that received only the final image. Evaluation used patient-grouped five-fold cross-validation over five seeds and three leakage-free encoder regimes. **Results:** On two matched binary tasks that hold the final image fixed—resolved versus absent (1→0 vs. 0→0) and onset versus persistent (0→1 vs. 1→1)—the single-image baseline performed at chance (AUROC 0.45 to 0.60), while the paired model scored well above it (paired difference +0.18 to +0.37 for resolution and +0.12 to +0.34 for onset; all significant by patient-level bootstrap after multiple-comparison correction), reaching a resolved-versus-absent AUROC of up to 0.87 (support devices). The benefit was stable across seeds, encoder regimes, projection changes, and follow-up intervals, and the CheXpert-derived transitions agreed with radiologist annotations (Cohen’s κ up to 0.97). **Conclusions:** A prior radiograph adds value whenever the final image alone underdetermines the transition, and they support pair-based designs for longitudinal change analysis.

## Simple Summary

Doctors who read chest X-rays almost always compare the new image with an earlier one to see whether a problem has appeared, improved, or stayed the same. Most artificial intelligence tools, however, look at only one image at a time, so they cannot tell whether a finding is new or has healed. In this study, we trained a model that looks at two chest X-rays of the same patient taken at different times and decides how specific findings, such as fluid around the lungs, have changed. Using more than 14,000 image pairs from a large public database, we show that the two-image model recognizes improvement and newly appearing findings far better than an equally trained one-image model, which often performs no better than guessing. These results support the construction of future medical imaging tools that, like radiologists, compare images over time rather than judging a single image in isolation.

## 1. Introduction

Radiological interpretation is comparative by nature. In clinical practice, a chest radiograph is rarely read on its own; when earlier imaging exists, the current study is compared with it to decide whether findings are new, worse, better, or unchanged. Reporting guidelines explicitly recommend this comparison and its documentation in the report [1]. In many situations, the relevant question is therefore not whether an abnormality is present, but whether it has changed over time. For outcomes such as clearing of pulmonary edema or a pleural effusion after treatment, this question needs temporal information: a current radiograph with clear lungs cannot show, by itself, what was present before. Dedicated temporal chest-imaging techniques, such as dynamic (functional) radiography, have also been developed to capture time-resolved information [2].

In contrast, much of the deep learning literature has treated chest radiography as a single-image classification problem. Large labeled resources such as CheXpert and MIMIC-CXR/MIMIC-CXR-JPG, distributed through the PhysioNet platform, have enabled models for image-level and finding-level classification [3,4,5,6], and several systems have reported performance comparable to radiologists for selected findings [7,8]. A single-image model, however, cannot tell whether an observed finding is new or persistent, nor can it establish that a previously present finding has been resolved. How much a model gains from a longitudinal image pair, and for which findings, therefore remains insufficiently characterized.

Longitudinal and comparative modeling has been studied in several medical imaging settings. Siamese networks have been used to grade disease severity and change in retinal photographs and knee radiographs [9], and dedicated methods have been proposed for interval change between sequential chest radiographs [10,11]. Temporal information from prior images and reports have also been used in vision-language modeling and report generation [12,13]. Siamese architectures are a natural choice for paired inputs because their two symmetric branches share the same weights and produce representations that can be compared directly [14]. Extending this idea to multi-finding chest radiograph progression raises three challenges. First, transition labels must be derived from per-image labels at two timepoints, which inherits label noise and creates strong class imbalance dominated by the absent-to-absent state. Second, onset and resolution are individually uncommon for many findings, which makes their evaluation difficult. Third, pretraining the encoder on the same corpus can leak information if images from test patients contributed to representation learning; patient-level separation is therefore essential [15]. Without controlling this overlap, gains attributed to longitudinal comparison are hard to separate from information introduced during pretraining.

In this study, we quantify the contribution of longitudinal pairing to finding-specific change detection on MIMIC-CXR under an experimental design that is robust to pretraining leakage. For each finding, the task is to predict one of four transitions (absent-to-absent, onset, resolved, or persistent) from the patient’s earliest and latest frontal studies within a bounded interval. A Siamese DenseNet-121 encodes the two images, and their representations are fused through concatenation and feature difference. A single-image baseline receives only the final radiograph, which isolates the contribution of the prior image. Evaluation uses patient-grouped cross-validation, and the whole comparison is repeated under three encoder initialization regimes without train/test patient overlap. Our contributions are four-fold:

- Quantifying the value of longitudinal pairing: on two matched tasks that hold the final image fixed, the single-image baseline is at chance while the paired model is well above it for resolution (1→0 vs. 0→0) and onset (0→1 vs. 1→1) alike (paired AUROC gains +0.18 to +0.37 and +0.12 to +0.34), showing that a prior radiograph helps whenever the final image alone underdetermines the transition.
- A leakage-controlled design: the comparison is repeated with three encoder regimes without train/test patient overlap, and Siamese resolution performance stays similar across them.
- Architecture-aligned interpretability: a change-pathway Grad-CAM isolates the feature-difference branch and complements conventional paired Grad-CAM.
- A reproducible benchmark: the full pipeline and the cross-validation fold definitions are made publicly available on GitHub (https://github.com/haeren/mimic-cxr-siamese).

The rest of the paper describes the data and methods in Section 2, presents the results in Section 3, discusses their interpretation and limitations in Section 4, and concludes in Section 5.

## 2. Materials and Methods

**Figure 1:**
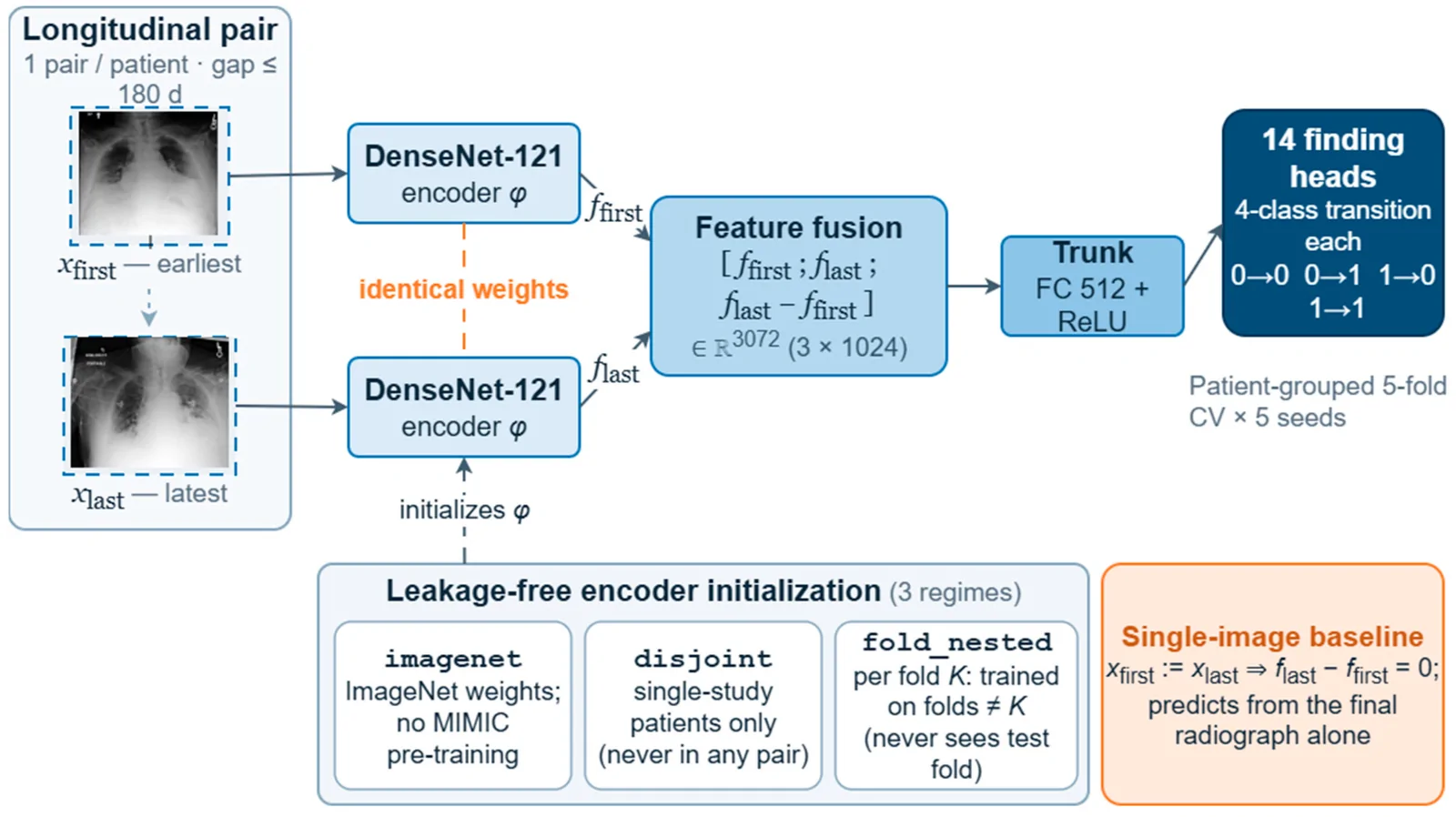
Overview of the longitudinal change-detection framework.

Figure 1 gives an overview of the experimental design. Each patient contributes one earliest–latest frontal pair within 180 days. A shared DenseNet-121 encodes the two images, and their features are fused (first image, last image, and their difference) before 14 finding-specific four-class transition heads. Three leakage-free encoder regimes (ImageNet, disjoint, and fold-nested) are evaluated, and a matched single-image baseline feeds the latest radiograph to both branches. Evaluation uses patient-grouped five-fold cross-validation across five random seeds.

### 2.1. Dataset, Pre-Processing, and Longitudinal Pairing

We used MIMIC-CXR-JPG v2.1.0 [5], including its metadata and CheXpert label files. The dataset is derived from the original MIMIC-CXR database [4] and was accessed through PhysioNet under its credentialed data use agreement [6]. Only frontal projections, antero-posterior (AP) and postero-anterior (PA), were included, because the findings studied here are assessed from frontal images. When a study contained several frontal images, one representative image was kept, with PA preferred over AP. Images were converted to RGB, padded to a centered square, and resized to 320 × 320 pixels. Labels came from the CheXpert automatic labeler distributed with the dataset [3,5], covering 14 observations with the states positive (1), negative (0), uncertain (−1), or unmentioned. We verified that patient identities did not overlap across the provided partitions, then recombined them and used patient-grouped cross-validation instead (Section 2.4).

For each patient with at least two distinct frontal studies, we built one longitudinal pair from the earliest and latest eligible studies. One pair per patient avoids repeated observations from the same person and matches the patient-level fold assignment. Temporal order was taken from the anonymized study dates, whose chronological order and intervals are preserved within each patient [5]. We examined pairing statistics under maximum follow-up windows of 90 and 180 days. The 90-day limit kept 11,664 of 30,742 eligible patients (37.9%; median interval 10 days), while the 180-day limit kept 14,043 patients (45.7%; median interval 16 days) and gave larger class support. The 180-day window was therefore used for all model training and evaluation; the 90-day window served only for preliminary characterization. A further 32,486 patients had only a single frontal study; these patients were used only for leakage-free encoder pretraining (Section 2.4) and never entered the change-detection dataset.

### 2.2. Transition Labels

For each finding, a four-class transition label was derived from the labeler states at the two timepoints. Each endpoint was treated as present when the raw label was 1 and not present when it was negative or unmentioned. It is important to note that, in radiology reports, an unmentioned finding is not necessarily radiographically absent; findings are typically reported when present or explicitly excluded, so silence usually—but not always—implies absence. In this cohort, the great majority of not-present endpoints were unmentioned rather than explicitly negated (ranging from about 82% to 100% across Tier-1 findings), so the 0→0 class in particular should be read as “negative or unmentioned” rather than be confirmed as negative. This is a known property of report-derived labels and is why the field-standard CheXpert convention treats unmentioned as negative; follow-up reports may additionally be selective, documenting a previously noted finding’s progression while omitting a stable known finding, so a follow-up is not always a complete reassessment. The resulting classes are defined in Table 1. All four classes are report-derived label transitions rather than radiologist-adjudicated events, and the “negative or unmentioned” reading of state 0 applies to every class that involves it: throughout the manuscript, onset (0→1) denotes a finding newly reported as present after being negative or unmentioned, and resolved (1→0) denotes a finding reported as present earlier and negative or unmentioned later. Because an unmentioned finding is not necessarily radiographically absent and follow-up reporting may be selective, a fraction of these label transitions may not correspond to true radiographic onset or resolution (see Section 4). For non-pathological observations such as No Finding and Support Devices, the terms likewise describe label-state changes rather than literal disease onset or resolution. If either endpoint was uncertain (−1), the transition was masked for that pair and excluded from training and evaluation. This conservative rule avoids inferring a change from an uncertain endpoint, but it also means that findings with high labeler uncertainty are judged only on their confident subsets (see Section 4).

The transition classes were highly imbalanced: absent-to-absent dominated every finding, while 0→1 and 1→0 were much rarer. Table 2 lists the per-finding support under the 180-day window; because every pair is a held-out test example exactly once, the pooled counts equal the effective evaluation support. Findings with at least 800 onset and 800 resolution transitions were defined as Tier-1 and form the primary analysis. The remaining findings are Tier-2 and are treated as secondary because their rare classes give wider confidence intervals. Pleural Other has too little support and is listed only for completeness.

### 2.3. Siamese Change Model and Single-Image Baseline

Let $x_{first}$ and $x_{last}$ denote the earliest and latest radiographs in a pair. A shared encoder φ maps each image to a 1024-dimensional feature vector obtained by global average pooling of the final convolutional block of a DenseNet-121 [16]:

(1) $$f_{first}=\varphi (x_{first}),\;\;f_{last}=\varphi (x_{last}).$$

The two representations are fused by concatenating the individual features with their difference:

(2) $$z=\left[f_{first};\;f_{last};\;f_{last}-f_{first}\right]\in R^{3072}.$$

The difference component explicitly represents change between the two timepoints, while the two original vectors keep the absolute state of each image. The fused representation passes through a shared trunk (a 512-unit fully connected layer, ReLU, and dropout) and then reaches 14 finding-specific heads, each producing four transition logits, so all findings share a common representation.

To measure the specific contribution of the prior radiograph, we built a matched baseline in which the final image is fed to both branches, i.e., $x_{first}∶=x_{last}$. Consequently,

(3) $$f_{last}-f_{first}=0,$$

and transition prediction must rely only on the final radiograph. This last-only baseline uses the same architecture, encoder, and training procedure; the only difference is the input. The performance gap between the Siamese and last-only models is the primary measure of the value of longitudinal pairing.

### 2.4. Cross-Validation and Leakage-Controlled Encoder Pretraining

We used patient-grouped five-fold cross-validation. Since each patient contributes exactly one pair, a seeded random partition assigned patients to five nearly equal, patient-disjoint folds of about 2808–2809 pairs. Every pair appears in a held-out fold exactly once, so pooled test predictions cover the full dataset. Fold membership was generated once, stored in a common fold map, and remained unchanged throughout the pipeline, including when deciding which patients were eligible for encoder pretraining.

To test whether the benefit of pairing depends on encoder pretraining, the shared encoder was initialized under three regimes, all without train/test patient overlap: (i) ImageNet, using ImageNet-pretrained DenseNet-121 weights [17,18] with no MIMIC-CXR pretraining; (ii) disjoint, pretrained only on the patients with a single frontal study, who cannot appear in the pair dataset; (iii) fold-nested, where a separate encoder was pretrained for each fold using only paired patients from the other folds. Encoder pretraining was a standard single-image multi-label task: a DenseNet-121 with a 14-output sigmoid head trained for up to 10 epochs with masked binary cross-entropy, AdamW (learning rate 2 × 10^−4^, warm-up then cosine decay, weight decay 10^−4^), gradient clipping at 5, positive-class weighting capped at 10, and mixed precision. Uncertain labels received a fixed soft target of 0.75, a variant of the U-Ones label-smoothing policy of Pham et al. [19] chosen because it gave the highest mean validation AUROC in a policy sweep. Only the convolutional backbone was transferred; the classification head was discarded. Leakage prevention was enforced programmatically: an assertion checked before training if the permitted pretraining patients did not intersect with the held-out set, and early stopping used a 10% patient-disjoint validation subset drawn only from the permitted population. In total, six MIMIC-pretrained encoders were built (one disjoint and five fold-nested), while the ImageNet regime needed none.

### 2.5. Training, Evaluation, and Interpretability

The change models were optimized with a masked, class-weighted cross-entropy loss. Class weights were the inverse class frequencies computed on the training folds only, normalized to a mean of one within each finding and capped at ten. Uncertain transitions were masked from the loss. Optimization used AdamW (initial learning rate 10^−4^, warm-up then cosine decay, weight decay 10^−4^), dropout 0.3, gradient clipping at 5, mixed precision, and a batch size of 32 for up to 12 epochs. Within each fold, 10% of training patients formed a patient-disjoint validation subset for early stopping and model selection, using Tier-1 macro-F1 as the criterion. Pretrained encoders stayed fixed across seeds; the five seeds (0, 26, 33, 42, and 99) affected only change-model training. The full experiment combined three encoder regimes, two input modes, and five seeds, giving 30 complete cross-validated runs.

Because absent-to-absent dominates every finding, overall accuracy was not used. We report per-class recall, macro-F1 over the four classes, and one-versus-rest AUROC for onset (0→1) and resolution (1→0). For each run, predictions from the five held-out folds were pooled. Because the one-versus-rest framing lets a current-image model exploit the dominant absent-to-absent class, we additionally define two matched binary discrimination tasks that isolate the value of the prior image: resolved-versus-absent (1→0 vs. 0→0) and onset-versus-persistent (0→1 vs. 1→1). For every finding and task, we report both AUROC and average precision (AUPRC), with the latter given the class imbalance. To quantify uncertainty at the level of patients rather than training runs, we averaged the softmax predictions of the five seeds per patient (an ensemble) and computed 95% confidence intervals by patient-level bootstrap resampling (2000 resamples). The Siamese and single-image models were resampled on the same patients so that the paired difference is preserved. Across the seven Tier-1 findings, per-finding *p*-values were corrected for multiple comparisons by the Holm and Benjamini–Hochberg procedures, and bootstrap *p*-values are floored at 1/(B + 1) and reported accordingly. Robustness to encoder pretraining was assessed descriptively across the three regimes.

For interpretability, two complementary Grad-CAM explanations [20] were generated from trained models on held-out pairs. Paired Grad-CAM propagates gradients for a selected finding and transition logit through the shared encoder to give one saliency map per image. Change-pathway Grad-CAM detaches the two absolute-state components of the fused representation so that gradients flow only through the $f_{last}-f_{first}$ difference pathway, isolating attribution linked to change. Because the two radiographs are not spatially registered, the per-image maps are shown side by side and are not subtracted; each map is min–max normalized to [0, 1] before overlay.

## 3. Results

### 3.1. Matched Binary Tasks Isolate the Contribution of the Prior Radiograph

The decisive test of whether the prior radiograph carries information is a comparison in which the single-image baseline sees exactly the final radiograph the paired model sees. We therefore evaluated two matched binary tasks. In resolved-versus-absent (1→0 vs. 0→0), where both cases end with the finding not reported as present (negative or unmentioned) in the final study, the single-image baseline performed at chance for every Tier-1 finding (AUROC 0.49 to 0.60), whereas the paired model reached 0.69 to 0.87. The paired-minus-baseline AUROC difference ranged from +0.18 to +0.37, and every 95% patient-level bootstrap interval excluded zero after Holm and Benjamini–Hochberg correction (Table 3). The gains were similar in average precision (AUPRC +0.12 to +0.40). Because the two cases differ only in the earlier label state, this isolates the contribution of the prior image: with the final radiograph held identical, only the model that also sees the earlier study can separate a finding labeled as resolved from one labeled as not present at both timepoints. The same pattern held for onset-versus-persistent (0→1 vs. 1→1), where both cases end with the finding reported as present: the baseline was again near chance (AUROC 0.45 to 0.53) and the paired model was well above it (+0.12 to +0.34 AUROC, all significant), because distinguishing a newly reported finding from one already reported as present likewise requires the earlier image. Prior imaging therefore adds value across both directions of label change whenever the final image alone underdetermines the transition.

**Figure 2:**
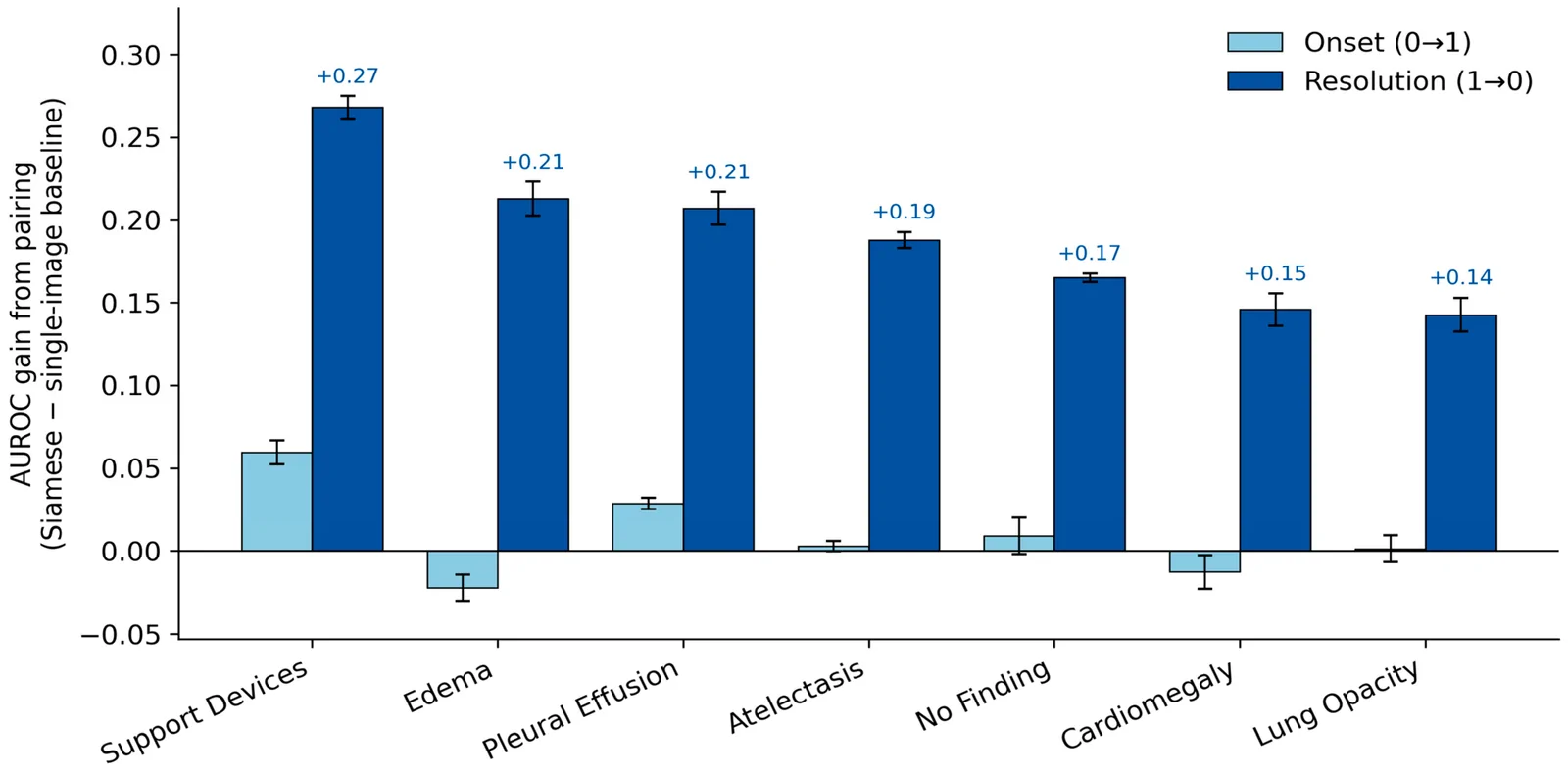
Differential effect of longitudinal pairing on onset and resolution detection. Bars show the mean change in one-versus-rest AUROC (Siamese − baseline) across five seeds for the seven Tier-1 findings under the fold-nested regime; error bars are 95% CIs. Gains are large and consistent for resolution (1→0) and small and mixed for onset (0→1). As explained in the text, this apparent asymmetry reflects the one-versus-rest evaluation framing rather than a limitation of the paired model: the dominant absent-to-absent class inflates the single-image baseline for onset. The matched onset-versus-persistent task (Table 3) removes this shortcut and shows a clear onset benefit.

In the one-versus-rest framing (refers to Table 4 and Figure 2), the apparent benefit of pairing looked strongly asymmetric: large for resolution (resolved-versus-rest AUROC +0.14 to +0.27) but small and mixed for onset (−0.02 to +0.06). This asymmetry is an artifact of the evaluation framing rather than a property of the model. In one-versus-rest, each transition is scored against all others, so the onset score is dominated by the many absent-to-absent cases, which the single-image baseline easily separates from the final radiograph alone; this inflates the baseline for onset and hides the benefit of the prior image. Removing that shortcut with the matched onset-versus-persistent task restores a clear onset benefit. Four-class macro-F1 also improved with pairing for every Tier-1 finding (paired differences +0.04 to +0.19).

### 3.2. Performance Is Stable Across Seeds and Encoder Regimes

The longitudinal model was highly reproducible across random seeds: for Tier-1 resolved-AUROC, the mean half-width of the 95% CI was 0.006, which was small relative to the gains in Table 3. Siamese performance was also consistent across the three leakage-free encoder strategies. As Table 5 shows, resolved-versus-rest AUROC values were closely matched across the ImageNet, disjoint, and fold-nested regimes, with a mean between-regime spread of only 0.020 across the seven Tier-1 findings. Similar performance was therefore obtained with an encoder never exposed to MIMIC-CXR, with an encoder pretrained on entirely separate patients, and with fold-specific encoders excluding the held-out patients. The disjoint regime was numerically higher for several findings, but the differences were small; we read Table 5 as descriptive robustness rather than as evidence that one regime is superior. Together, these results indicate that resolution performance is not driven by a particular pretraining strategy or by train/test patient overlap.

### 3.3. Projection Change Does Not Explain the Effect

A concern with longitudinal comparison is that patients often move from portable AP to PA imaging as they improve, so a projection change could coincide with resolution. Using the DICOM view position for each paired image, we re-evaluated the resolved-versus-absent task on the 9806 pairs whose projection did not change (both PA or both AP; a well-powered majority of the 14,043 pairs). The Siamese–baseline gain was essentially unchanged from the full cohort (AUROC differences +0.19 to +0.45 versus +0.18 to +0.37), and the single-image baseline remained at chance (AUROC 0.41 to 0.59), so projection change cannot account for the effect. Where a projection effect was detectable at all (support devices, baseline AUROC 0.41 on same-projection versus 0.65 on changed-projection pairs), it inflated the single-image baseline rather than the paired model, which makes the reported gains conservative. Cardiomegaly, the finding most plausibly sensitive to projection, showed almost no difference between same- and changed-projection pairs.

### 3.4. The Benefit Is Stable Across Follow-Up Intervals

Pairs span a range of inter-study intervals, so we stratified both matched tasks into 0→30-, 31→90-, and 91→180-day bins (8728/2936/2379 pairs). The resolution benefit was stable across all bins (for example, the resolved-versus-absent AUROC difference for atelectasis was +0.235, +0.251, and +0.254 across the three bins), with a slight increase at longer gaps for several findings. The onset benefit was present in every bin but declined modestly at the longest interval for some findings (support devices onset AUPRC +0.319, +0.187, +0.154 across bins; atelectasis +0.187, +0.158, +0.108), consistent with an older prior image being less informative about a newly reported finding. Rare-transition cells thinned in the 91→180-day bin (for example, edema onset, *n* ≈ 111→136), where intervals are correspondingly wider.

### 3.5. Transition Labels Agree with Radiologist Annotations

To check that the CheXpert-derived transitions reflect radiographic change rather than labeler noise, we compared them with the public single-radiologist reference annotations for the MIMIC-CXR test set (687 studies; the reference names Lung Opacity as “Airspace Opacity”, which we mapped). Of our pairs, 101 endpoint studies and 28 complete pairs overlapped with the reference. At the endpoint level, agreement was substantial to almost perfect for six of seven Tier-1 findings (Cohen’s κ = 0.68 to 0.97), with No Finding being the weakest (κ = 0.46), a noisy derived meta-label. For the 28 complete pairs, the four-class transition agreed with the radiologist-derived transition at κ = 0.58 to 0.94. Resolved-class agreement was high for most findings—κ = 1.0 for cardiomegaly, atelectasis, and pleural effusion, 0.88 for edema, and 0.70 for lung opacity, and it was lower for no finding (0.63) and support devices (0.51); these estimates each rest on only a handful of cases. These results indicate the transitions are radiologist-concordant, subject to the single-reader reference, the modest overlap, and the fact that this validates the direction of change rather than the exact four-class scheme; full multi-reader adjudication is reserved for future work.

### 3.6. Tier-2 Findings

The less frequent Tier-2 findings showed the same general pattern of improved resolution detection, but with clearly wider confidence intervals given their lower support (Table 2). We therefore treat these results as secondary and avoid strong finding-specific conclusions. Several Tier-2 findings, notably Consolidation and Enlarged Cardiomediastinum, had very few persistent cases, which added variability to their four-class macro-F1 estimates.

### 3.7. Interpretability

**Figure 3:**
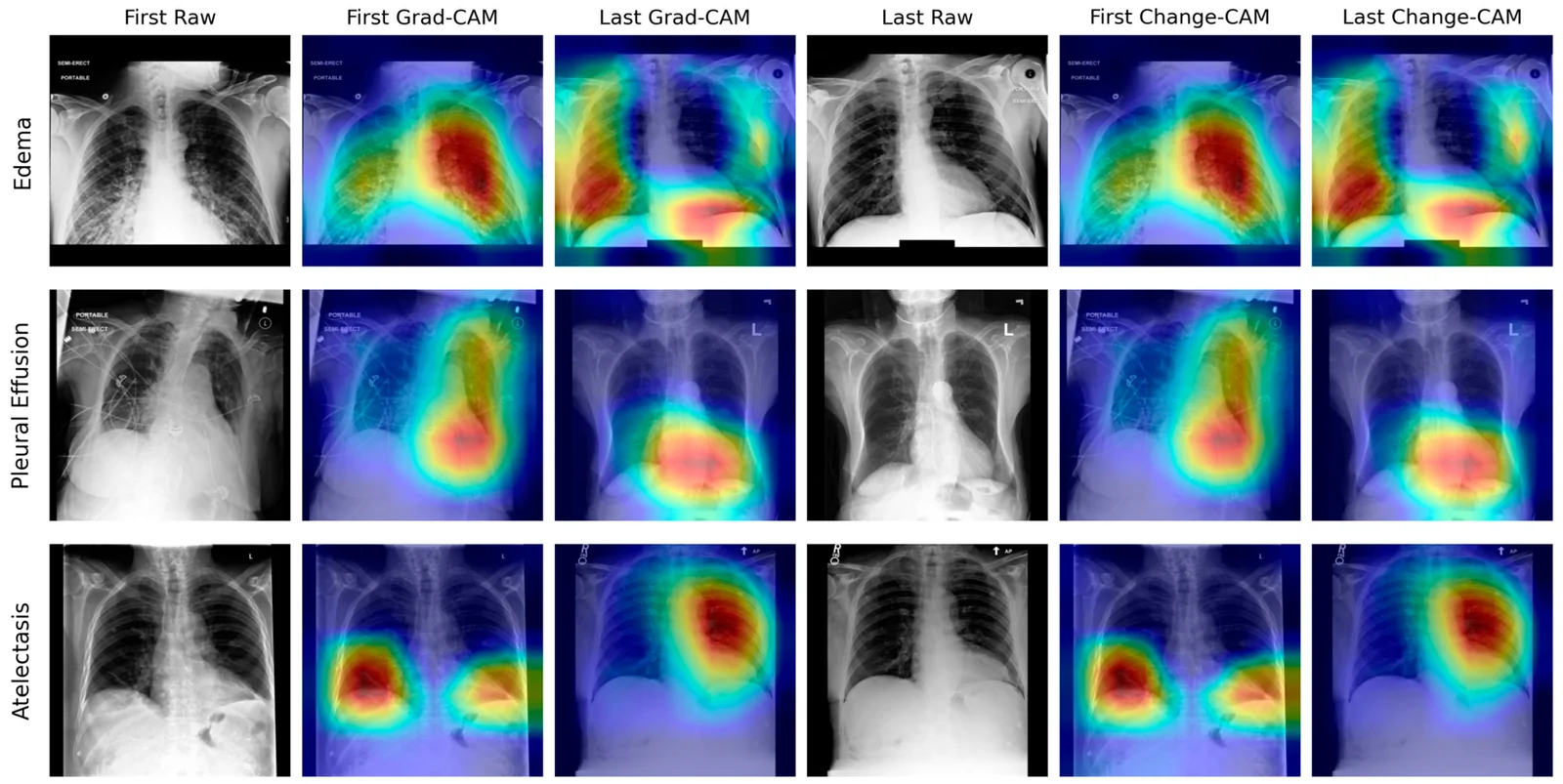
Paired and change-pathway Grad-CAM for representative resolution (1→0) examples: edema (**top**), pleural effusion (**middle**), and atelectasis (**bottom**). In each row, the left panel shows the earlier radiograph, paired Grad-CAM on the first and last images, and the later radiograph; the right panel shows the corresponding change-pathway maps, restricted to the $f_{last}-f_{first}$ difference pathway. The selected paired and change-pathway maps essentially emphasize the same regions at corresponding timepoints. Heatmaps are independently min–max normalized and were not validated against expert annotations; examples come from held-out pairs (seed 0, fold 1).

Paired and change-pathway Grad-CAM produced spatially localized saliency in the selected high-confidence resolution examples (Figure 3). For edema, pleural effusion, and atelectasis, the paired maps emphasized localized regions on the earlier radiograph that were less prominent on the later one, and the paired and change-pathway maps looked very similar at corresponding timepoints, even though the change-pathway attribution is restricted to the $f_{last}-f_{first}$ difference pathway. This similarity is reported descriptively. However, no expert region annotations were available, so these locations were not independently validated, and the maps should be read as qualitative attributions rather than validated anatomical grounding.

## 4. Discussion

Framing chest radiograph interpretation as a comparison between a patient’s prior and current examinations, rather than as classification of a single image, produced large and reproducible improvements in change detection. The clearest evidence comes from two matched tasks in which the single-image baseline sees exactly the final radiograph the paired model sees: in resolved-versus-absent (1→0 vs. 0→0) and onset-versus-persistent (0→1 vs. 1→1), the baseline performed at chance (AUROC 0.45 to 0.60) while the paired model scored well above it (paired difference +0.18 to +0.37 and +0.12 to +0.34, respectively; all significant by patient-level bootstrap after correction). Because the two cases in each task share an identical final image, this isolates the contribution of the prior radiograph and shows that its value is broad: it helps whenever the final image alone cannot reveal the history of a finding. An apparent asymmetry in an earlier analysis—a large-resolution benefit but little-onset benefit under one-versus-rest scoring (Table 4, Figure 2)—proved to be an artifact of that framing. In one-vs-rest, the many absent-to-absent cases let the single-image baseline separate onset from the rest using the final image alone, which inflates the onset baseline and hides the benefit of the prior image. The benefit was reproducible across five seeds and three leakage-free encoder regimes, including an ImageNet-only encoder never exposed to MIMIC-CXR, so it is not explained by pretraining or by patient overlap. It also persisted on same-projection pairs and across follow-up intervals, and the CheXpert-derived transitions agreed with radiologist reference annotations (endpoint κ up to 0.97; high agreement on the resolved class), supporting the reliability of the labels underlying these findings.

These results agree with radiological practice, where comparison with prior studies is an established part of interpretation [1]. They suggest that systems intended for longitudinal monitoring—for example, detecting interval resolution of edema or pleural effusion after treatment, or the new appearance of a finding—may benefit from explicitly using prior imaging rather than only the current radiograph, and that purely cross-sectional evaluation does not assess this capability. The benefit is not perfectly uniform: for a few findings (for example, cardiomegaly and lung opacity), the onset gain in average precision is smaller, and the baseline is already comparatively strong, so the advantage is largest where the two endpoints are most easily confused. The absolute performance also tempers clinical claims: Tier-1 resolved-versus-absent AUROC ranged from about 0.69 to 0.87, so the present system is a controlled demonstration of the value of pairing rather than a deployable change-detection tool, and the main contribution is to quantify, under controlled conditions, how much information a prior radiograph carries about the transition a finding has undergone.

Pairwise and longitudinal modeling has been explored before. Siamese architectures have been applied to longitudinal medical image comparison [9], Oh et al. introduced geometric correlation maps for interval change on chest radiographs [10], CheXRelNet added anatomical information for longitudinal pathology relationships [11], and CheXRelFormer used a Siamese hierarchical Transformer for progression classification [21]. Similarly, Yu et al. developed a twin network that classifies anatomy-specific progression into four categories from weakly supervised, report-derived labels [22]. Like the other studies above, this work centers on building a progression classifier, whereas our aim is to quantify how much the prior image itself contributes by comparing a paired model against a matched single-image baseline under leakage control, rather than to propose a new progression architecture. Temporal information has also entered vision-language modeling: BioViL-T learns temporal representations from prior examinations [12], TempA-VLP extends this with temporal-aware pretraining [23], recent work addresses sensitivity to temporal order [24], MI-CXR benchmarks reasoning over multiple examinations [25], and longitudinal context has been used in report generation [13]. Change detection between co-registered images is also a long-standing task in other imaging domains, such as remote sensing [26], where robustness and generalization across acquisition conditions are likewise central concerns. Against this background, our contribution is neither a new pairwise architecture nor the first longitudinal application. We instead answer a more specific question: how much predictive value does the prior radiograph add over the current image alone, and how does that value differ across transitions? The matched last-only baseline isolates this contribution, the four-class formulation separates onset from resolution instead of collapsing change into one category, and the explicit control of patient overlap during pretraining, including an ImageNet-only regime and fold-nested pretraining, separates the effect of temporal information from a possible pretraining confound. We therefore position this study as a controlled, leakage-aware quantification rather than a novelty claim.

Transition labels were built from the CheXpert automatic labeler [3,5], so an observed 0→1 or 1→0 transition may reflect either true radiographic change or labeler inconsistency between examinations; no radiologist-adjudicated labels were available. Because unmentioned findings were treated as not present, the ambiguity of state 0 applies to all transition classes, not only 0→0: an apparent resolution (1→0) may include findings that remained visible but were unreported at follow-up, and an apparent onset (0→1) may include findings present but unmentioned at baseline, particularly since follow-up reports can be selective. The transitions studied here are therefore report-derived label transitions, and the reported gains quantify the value of the prior image for discriminating those transitions rather than adjudicated radiographic change. We note that in the resolved-versus-absent task, the single-image baseline performed at chance on the final radiograph. If a substantial share of 1→0 cases still carried a visible finding mislabeled as not present, the final image alone would have been informative, so this pattern is at least consistent with the label transitions predominantly reflecting genuine change, though it remains indirect evidence. Masking uncertain endpoints reduces this risk but means that findings with high uncertainty, such as pneumonia and edema, are evaluated only on their confident subsets, which may not represent all cases. Each patient contributes a single earliest–latest pair within 180 days, so intermediate trajectories, repeated follow-ups, the magnitude of change, longer-term progression, lateral views, and clinical events between examinations are all outside the scope of this analysis, and patients with a single frontal study could not contribute.

The Grad-CAM analyses are qualitative: localization faithfulness was not quantitatively assessed because expert region annotations were unavailable. Moreover, the displayed maps are drawn from selected high-confidence resolution examples and are not a random sample, so they illustrate model behavior rather than provide an unbiased assessment of localization quality. A blinded radiologist review of a larger, representative sample would be required for that purpose. Finally, all experiments used MIMIC-CXR, a single-center dataset [4]. External validation on an independent longitudinal cohort, ideally with radiologist-adjudicated change labels, is needed to confirm that the observed effects generalize across institutions, populations, and imaging systems.

## 5. Conclusions

Chest radiographs are commonly read in relation to prior examinations, while many computational methods still operate on single images. Using a leakage-controlled design on MIMIC-CXR, we found that modeling a longitudinal radiograph pair produced large and reproducible improvements in finding-specific change detection, with prior imaging adding value across both directions of label change—resolution and onset alike—whenever the final image alone underdetermines the transition. An apparent onset–resolution asymmetry under one-versus-rest scoring proved to be an artifact of that evaluation framing. The gains were stable across random seeds and across three encoder-pretraining regimes without train/test patient overlap, including an ImageNet-only regime. We make no claim of clinical deployability: this is a controlled demonstration of the value of prior imaging, and independent external validation with radiologist-adjudicated change labels remains necessary before any deployment.

These results quantify how much a prior radiograph contributes to change detection, as well as supporting comparative, pair-based formulations when the goal is to characterize change over time. The pipeline and fold definitions will be released to provide a reproducible framework. Future work will focus on radiologist-adjudicated or region-grounded change labels, extension of the two-image design to multi-visit sequences with explicit time intervals, dedicated pairwise tasks such as 1→0 vs. 0→0, and spatial registration of paired radiographs.

## Figures and Tables

**Table 1:** The four transition classes derived from paired finding labels at the two timepoints.

Code	Transition	Meaning
0	0→0	Absent (negative or unmentioned) at both studies
1	0→1	Onset (newly reported present; previously negative or unmentioned)
2	1→0	Resolved (no longer reported present; negative or unmentioned at follow-up)
3	1→1	Persistent

**Table 2:** Per-finding transition support within the 180-day follow-up window among 14,043 pairs.

Finding	Tier	Onset (0→1)	Resolved (1→0)	Persistent (1→1)	Absent (0→0)	Masked
Lung Opacity	1	2106	2273	1215	7924	525
Support Devices	1	2409	1893	1686	8024	31
No Finding	1	1893	2307	2062	7781	0
Atelectasis	1	2067	1597	1026	7771	1582
Cardiomegaly	1	2107	1456	889	8843	748
Pleural Effusion	1	2463	1012	1419	8229	920
Edema	1	1012	848	471	10,018	1694
Pneumonia	2	738	816	179	9721	2589
Consolidation	2	638	437	83	12,282	603
Enlarged Cardiomediastinum	2	415	452	39	12,047	1090
Pneumothorax	2	327	384	149	13,035	148
Lung Lesion	2	258	418	158	12,999	210
Fracture	2	196	314	118	13,356	59
Pleural Other	-	117	91	19	13,686	130

**Table 3:** Discrimination on the two matched binary tasks under the fold-nested regime, Siamese versus single-image baseline. Values are the five-seed ensemble AUROC and AUPRC for each model; ∆ is the paired Siamese–baseline difference with a 95% patient-level bootstrap interval (2000 resamples), all significant after Holm and Benjamini–Hochberg correction. Baseline AUROC near 0.50 indicates the final image alone cannot separate the two classes.

Finding	Task A. Resolved Versus Absent (1→0 vs. 0→0)							
	Siamese AUROC	Baseline AUROC	∆ AUROC	95% CI	Siamese AUPRC	Baseline AUPRC	∆ AUPRC	95% CI
No Finding	0.754	0.490	+0.264	[+0.251, +0.277]	0.506	0.242	+0.264	[+0.246, +0.282]
Support Devices	0.867	0.496	+0.371	[+0.356, +0.386]	0.632	0.228	+0.404	[+0.382, +0.424]
Cardiomegaly	0.740	0.564	+0.175	[+0.160, +0.192]	0.296	0.176	+0.119	[+0.100, +0.138]
Lung Opacity	0.685	0.494	+0.191	[+0.176, +0.206]	0.348	0.227	+0.121	[+0.106, +0.137]
Atelectasis	0.751	0.506	+0.245	[+0.229, +0.261]	0.368	0.178	+0.191	[+0.170, +0.211]
Pleural Effusion	0.805	0.533	+0.271	[+0.253, +0.288]	0.384	0.129	+0.254	[+0.225, +0.283]
Edema	0.845	0.604	+0.241	[+0.224, +0.257]	0.343	0.114	+0.230	[+0.201, +0.261]
**Finding**	**Task B. Onset Versus Persistent (0→1 vs. 1→1)**							
	**Siamese** **AUROC**	**Baseline** **AUROC**	**∆** **AUROC**	**95% CI**	**Siamese** **AUPRC**	**Baseline** **AUPRC**	**∆** **AUPRC**	**95% CI**
No Finding	0.725	0.490	+0.235	[+0.216, +0.255]	0.717	0.495	+0.223	[+0.200, +0.244]
Support Devices	0.864	0.522	+0.342	[+0.325, +0.360]	0.897	0.616	+0.281	[+0.262, +0.300]
Cardiomegaly	0.671	0.533	+0.138	[+0.114, +0.162]	0.818	0.727	+0.091	[+0.071, +0.110]
Lung Opacity	0.613	0.498	+0.115	[+0.095, +0.134]	0.746	0.647	+0.099	[+0.080, +0.117]
Atelectasis	0.674	0.451	+0.224	[+0.201, +0.244]	0.819	0.644	+0.175	[+0.156, +0.193]
Pleural Effusion	0.808	0.501	+0.307	[+0.288, +0.324]	0.876	0.657	+0.219	[+0.201, +0.237]
Edema	0.789	0.515	+0.274	[+0.242, +0.307]	0.888	0.698	+0.190	[+0.160, +0.216]

**Table 4:** Effect of longitudinal pairing on transition detection for Tier-1 findings under the fold-nested regime. Values are one-vs-rest AUROC averaged over five seeds; ∆ is the paired mean difference (Siamese − baseline) with the paired-*t p*-value.

Finding	Onset (0→1) AUROC				Resolved (1→0) AUROC			
	Siamese	Baseline	∆	*p*	Siamese	Baseline	∆	*p*
Support Devices	0.826	0.767	+0.059	2.1 × 10^−5^	0.843	0.575	+0.268	4.2 × 10^−8^
Edema	0.798	0.820	−0.022	1.5 × 10^−3^	0.816	0.603	+0.213	5.4 × 10^−7^
Pleural Effusion	0.829	0.800	+0.029	2.2 × 10^−5^	0.784	0.577	+0.207	5.5 × 10^−7^
Atelectasis	0.743	0.740	+0.003	6.0 × 10^−2^	0.725	0.537	+0.188	4.5 × 10^−8^
No Finding	0.698	0.689	+0.009	8.3 × 10^−2^	0.743	0.578	+0.165	6.0 × 10^−9^
Cardiomegaly	0.681	0.694	−0.013	2.6 × 10^−2^	0.708	0.562	+0.146	2.0 × 10^−6^
Lung Opacity	0.670	0.669	+0.001	6.8 × 10^−1^	0.660	0.517	+0.143	2.6 × 10^−6^

**Table 5:** Robustness of Siamese resolution detection across encoder regimes. Resolved-versus-rest AUROC is mean ± 95% CI across five seeds.

Finding	ImageNet	Disjoint	Fold-Nested
Support Devices	0.817 ± 0.005	0.837 ± 0.005	0.843 ± 0.004
Edema	0.818 ± 0.002	0.828 ± 0.015	0.816 ± 0.008
Pleural Effusion	0.778 ± 0.006	0.796 ± 0.009	0.784 ± 0.003
No Finding	0.736 ± 0.003	0.757 ± 0.005	0.743 ± 0.006
Atelectasis	0.708 ± 0.008	0.731 ± 0.008	0.725 ± 0.003
Cardiomegaly	0.690 ± 0.006	0.713 ± 0.009	0.708 ± 0.008
Lung Opacity	0.651 ± 0.005	0.665 ± 0.006	0.660 ± 0.008

## Data Availability

The MIMIC-CXR-JPG v2.1.0 dataset is available to credentialed researchers through PhysioNet [5]. Access requires PhysioNet credentialing, completion of the required CITI Data or Specimens Only Research training, and acceptance of the PhysioNet Credentialed Health Data Use Agreement [5]. The source code and exact cross-validation fold definitions used in this study are publicly available on GitHub (https://github.com/haeren/mimic-cxr-siamese, accessed 8 September 2026).

## Abbreviations

The following abbreviations are used in this manuscript:

AP	Antero-posterior
AUROC	Area under the receiver operating characteristic curve
CI	Confidence interval
CXR	Chest radiograph (chest X-ray)
Grad-CAM	Gradient-weighted class activation mapping
PA	Postero-anterior
